# Integrating intuitionistic fuzzy and MCDM methods for sustainable energy management in smart factories

**DOI:** 10.1371/journal.pone.0315251

**Published:** 2025-01-14

**Authors:** Mohd Anjum, Naoufel Kraiem, Hong Min, Yousef Ibrahim Daradkeh, Ashit Kumar Dutta, Sana Shahab

**Affiliations:** 1 Department of Computer Engineering, Aligarh Muslim University, Aligarh, India; 2 College of Computer Science, King Khalid University, Abha, Saudi Arabia; 3 School of Computing, Gachon University, Seongnam, Republic of Korea; 4 Department of Computer Engineering and Information, College of Engineering in Wadi Alddawasir, Prince Sattam Bin Abdulaziz University, Al-Kharj, Saudi Arabia; 5 Department of Computer Science and Information Systems, College of Applied Sciences, AlMaarefa University, Ad Diriyah, Riyadh, Kingdom of Saudi Arabia; 6 Department of Business Administration, College of Business Administration, Princess Nourah Bint Abdulrahman University, Riyadh, Saudi Arabia; Ankara Yildirim Beyazit University / Worcester Polytechnic Institute, TÜRKIYE

## Abstract

Improving energy efficiency is crucial for smart factories that want to meet sustainability goals and operational excellence. This study introduces a novel decision-making framework to optimize energy efficiency in smart manufacturing environments, integrating Intuitionistic Fuzzy Sets (IFS) with Multi-Criteria Decision-Making (MCDM) techniques. The proposed approach addresses key challenges, including reducing carbon footprints, managing operating costs, and adhering to stringent environmental standards. Eight essential criteria are identified, such as the use of renewable energy, the efficiency of production, and the health and safety of workers, to evaluate energy performance. Using the entropy method for criterion weighting and the CRADIS technique for alternative ranking, we prioritize a range of energy-efficient solutions. The novelty of our approach lies in its comprehensive assessment of complex real-world energy management scenarios within smart factories, offering a robust and adaptable decision-support tool. Our empirical results, validated through sensitivity analysis, show that alternative 5 delivers the most significant improvement in energy efficiency. This study provides valuable information for industry practitioners seeking to transition to more sustainable production methods and supports the broader sustainability agenda.

## 1 Introduction

In the age of Industry 4.0, smart factories have become the cornerstone of contemporary production due to the spread of cutting-edge technologies such as the Internet of Things (IoT), artificial intelligence (AI), and big data analytics [1]. These factories are typified by traits such as self-optimization in performance, adaptability to changing conditions, and autonomy in the management of the whole process of production. One way to characterize a smart factory would use cyber-physical systems to track real-world activities, digitally replicate those actions, and make autonomous choices. The IoT allows these networks to communicate and collaborate in real-time with individuals as well as with each other [2]. As a result, information may now move freely throughout the whole production network. Increasing productivity and automating monotonous tasks are only some of the many benefits of smart factories. They enable producers to respond swiftly to market and client expectations changes because of the additional flexibility they offer. In addition, they improve worker safety by automating risky operations, minimizing downtime by anticipating maintenance needs, and improving product quality through precise management and process monitoring [3]. The administration and optimization of complex systems, which is a task that usually fails when standard methods are utilized, is one of the activities that have significant advantages.

To address the global issues of resource depletion, environmental degradation, and climate change, sustainable manufacturing techniques must be put into place [4]. In the industrial industry, sustainability is much more than a passing trend. Some sustainable production goals are reducing energy and resource usage, protecting communities and workers from harm, and minimizing negative environmental repercussions. It includes various actions, such as trash reduction, recycling, resource efficiency, and the use of renewable and environmentally friendly energy sources. As stated in [5]. Energy efficiency plays a critical role in the industrial sector in lessening the financial strain on businesses and the detrimental effects on the environment. Both the bottom line and the emissions of greenhouse gases are impacted by the amount of energy used in the manufacturing process. Consequently, it makes sense to prioritize improving energy efficiency from both a financial and environmental standpoint. Numerous strategies, including optimizing machine utilization, employing energy-efficient technologies and procedures, and implementing advanced energy management systems, may improve energy efficiency in smart factories [6].

The Sustainable development goals of The United Nations might be greatly advanced by integrating sustainable practices into smart factories, especially in climate action and responsible production and consumption [7, 8]. Manufacturers can create a sustainable ecosystem that lowers their environmental footprint while increasing productivity by using the potential of intelligent technology. Due to the inherent imprecision and subjective evaluations that come with considering a large number of elements, standard decision-making techniques are typically inadequate. IFS is now found to be advantageous. IFS, an extension of fuzzy sets, offers a more thorough framework by incorporating reluctance, membership, and non-membership levels into its creation. Complex contexts like smart factories are especially well-suited for MCDM because they provide a more flexible and nuanced portrayal of uncertainty. This is because they give a more realistic depiction of uncertainty.

This study aims to look into potential ways that sustainable smart factories might improve energy efficiency from the perspective of Intuitionistic Fuzzy (IF). We will be able to achieve our goal of creating a decision-support system that can rank and assess different options for raising energy efficiency levels by utilizing MCDM methodologies inside this framework. This approach, which aligns with broader sustainability goals and considers both the operational and technological elements of energy management, guarantees a significant advancement in creating environmentally friendly manufacturing processes. An overview of the areas that will be covered in this article is provided below: This study is divided into the following sections: a comprehensive literature review on smart factories, manufacturing sustainability, and the application of IFS in MCDM; a methodology outlining criteria selection, alternative identification, and MCDM technique application within an IF environment; a case study demonstrating the practical implementation of our proposed approach; analysis and discussion of the findings; and, finally, conclusions emphasizing the contributions, implications, and future directions for research.

## 2 Literature review

At times, understanding the complex manner in which professional information is presented may be challenging. According to Atanasov [9], the idea of IFS was first proposed for discussion. These sets build upon the concept of fuzzy sets, initially proposed by Zadeh [10]. When handling ambiguous circumstances, the IFS, which employs a “membership degree” and a “non-membership degree” is seen to be superior to Zadeh’s fuzzy sets. Its use has been widespread since its inception in many sectors, such as pattern recognition, market trend forecasting, and strategy formulation, to mention a few. The issue of how to gather data most efficiently has emerged as one of the most important challenges facing IFS MCDM research. Many researchers have approached this issue from different perspectives to guarantee the accuracy of the findings. Xu [11] presented a few fundamental mathematical methods to design Intuitionistic Fuzzy Numbers (IFNs). Xu and Yager [12] also described several significant geometric methods for MCDM that utilize IFNs.

Wu et al. [13] state that the researchers managed the data collected in various settings and at different times efficiently by using various dynamic approaches. Rahman and Muhammad [14] introduced aggregation operators. This study presented a significant enhancement in the modeling of fuzzy environments, where decision-making involves complex interrelated criteria. Yang et al. [15] concentrated on real-time aggregation management of power-to-heat loads in a virtual power plant. To maximize interactions between the virtual power plant operator and specific consumers and achieve energy efficiency and system stability, their research focused on the real-time coordination of power-to-heat loads. A variety of procedures have also been created by other scientists [16–18], such as the IF-point methods, the generalized IF-weighted techniques, and the IF-Choquet integral methods. Wu et al. have discovered two other approaches that include several sources. The IF-Bonferroni mean and the IF Aggregation Operators with weighted vectors are two examples of these techniques. Furthermore, in their separate studies, Wu and Wang [19] and Xu and Wang [20] introduced unique strategies that may be activated by impulse functions. Martin and Edalatpanah [21] introduced the extended fuzzy ISOCOV methodology for nanomaterial selection, which uses performance measures to rank and select appropriate nanomaterials, a crucial task in the engineering and manufacturing sectors.

Zeng and Su have created several MCDM methodologies [22–24]. IF-ordered weighted distance and IF-hybrid weighted distance are two of these methods. In their separate publications, Yu [22] and Wu [25] introduced geometric techniques and an IF-prioritized average. Other people submitted further recommendations in a similar vein. Some of the latest ones were even created by Yu [26]. Two examples are the “IF-geometric weighted Heronian mean”, and the “IF-geometric Heronian mean.” IF-probabilities are the basis for several novel techniques developed by Merigo and Wei [27]. Ma et al. [28] highlighted how price fluctuations affect customer choices. Their work illustrates the use of smart technology to improve energy efficiency by modeling dynamic pricing in the energy market and suggesting management solutions that balance energy usage while minimizing costs for residential buildings.

Weight computation must never be taken lightly since it is a basic and crucial step in disaster management. The entropy method, or EM for short, is a well-known and extensively studied weight model. The EM offers several advantages over other weighting models, the most important of which is that it increases the overall impartiality of the evaluation findings by preventing human biases from influencing the indicators’ weight. EM assesses the value of everything by measuring the degree of differentiation. More information may be extracted from an index with a higher degree of differentiation when the measured value’s dispersion is larger. Moreover, a significant amount of weight must be given to the index, and vice versa. Zhu et al.[29] essay explores the usefulness of the entropy technique in the decision-making process. Ma et al. [30] examined how demand response in smart grids may be accomplished via relaying-assisted communications. Focusing on cost modeling, game strategies, and algorithms, their research offers fresh ideas for communication optimization for efficiently regulating the consumption of energy. It has been applied in many different contexts, including the creation of mechanical products [31], stock investments [32], assessment of vulnerability to environmental risks [33, 34], and water quality analysis.

A unique technique to output synchronization was developed by Wang et al. [35] for wide-area heterogeneous multi-agent systems operating on intermittent clustered networks. The objective of this study is to improve system resilience by resolving the problems caused by network clusters and communication delays, and by putting forward a novel synchronization method for large-scale power systems. EM has also been used by investigators to look into SA. Liang and Wang [36] presented a proposal for an EM-based decision support system that aims to help online shoppers find items that interest them. As a result, this provided a methodical way to assess how well companies performed regarding sustainability. Pajić et al. [37] employed an FMEA combined with the QFD methodology to address risks in distribution processes. Their approach effectively assesses potential failures in the distribution chain and prioritizes corrective measures to mitigate risks.

The fuzzy TRUST CRADIS technique was utilized by Puška et al. [38] to choose sustainable suppliers in the agriculture industry. This approach is very significant because it makes it possible to assess and select providers under ambiguous circumstances by using sustainability standards. The CRADIS approach, an abbreviation for compromise ranking of alternatives from distance to Ideal Solution, was developed by Chakraborty [39]. Duan et al. [40] proposed an initialization-free distributed method for dynamic economic dispatch in microgrids, enabling real-time energy management. Their approach maximized the economic dispatch process while maintaining system stability, which is critical for microgrids that perform well under dynamic settings. Puška et al. [41] used the principles of entropy and CRADIS to assess the knowledge economies inside the European Union. This study illustrated how CRADIS may be effectively implemented in complicated decision-making scenarios by rating nations based on their global knowledge index.

The Fermatean fuzzy aggregation operators [42], are presented an analysis of their application and contribution. Fuzzy CRADIS and CRITIC approaches were used by Puška et al. [43] to evaluate the pear variety market in Serbia. The objective of this application was to assess and investigate several pear types using various criteria to illustrate the adaptability of CRADIS in agricultural markets. Abid and Saqlain [44] explored the integration of edge cloud computing and deep learning in the context of China’s international trade and investment. Wang et al. [45] have introduced a sophisticated spherical fuzzy CRADIS technique for assessing occupational hazards during natural gas pipeline building. This method is successful and is based on the Fine-Kinney framework. Fuzzy logic is included in this method to help handle uncertainties that come up during risk assessment. Feng et al. [46] analysed the life cycle cost of electricity production using underground coal gasification with carbon capture and storage. Their research assesses the economic feasibility of carbon capture and storage in coal gasification projects, offering information on the cost-effectiveness and long-term viability of incorporating carbon capture and storage into energy production.

Puška et al. [47] created a decision framework using a hybrid enhanced fuzzy SWARA and fuzzy CRADIS technique to choose distribution centre sites. By showing how CRADIS can optimise distribution network decisions in the face of uncertainty, this study illustrated the tool’s value in logistics. For application in an image and fuzzy environment, Yuan et al. [48] suggested applying the CRADIS approach together with a unique distance measure. This approach expands CRADIS’s capabilities to handle image fuzzy sets and improves its efficacy in decision-making under complicated and perplexing data conditions. Abbasi et al. [49] provide a comprehensive analysis of sustainable supply chain performance during this crisis, focusing on a real-life case study. Abbasi et al. [50] provide a comprehensive analysis of sustainable supply chain performance during this crisis, focusing on a real-life case study. The use of interval-valued picture fuzzy uncertain linguistic Dombi operators in industrial fund selection [51].

Altıntaş [52] performed a study based on the LOPCOW-based CRADIS approach to evaluate the prosperity performances of the G7 countries. This study aimed to assess the governments of the G7 countries based on their various prosperity indices by using CRADIS in global economic evaluations. The MCDM model for evaluating road section safety [53], and the application of edge cloud computing and deep learning for risk assessment in China’s international trade Puška et al. [54] suggested a hybrid MCDM approach to select ecologically friendly suppliers in agricultural scenarios when parameters are unknown. This model incorporates several fuzzy methodologies to strengthen the decisions on supplier selection. These are but a few of the instances. Research in this area has shown a wide range of strategies for managing risk and coming to judgments. The study’s conclusions will probably be very helpful when making judgments on renewable energy. Advanced decision-making techniques, such as Pythagorean fuzzy Hamacher aggregation operators [55].

**Fig 1:**
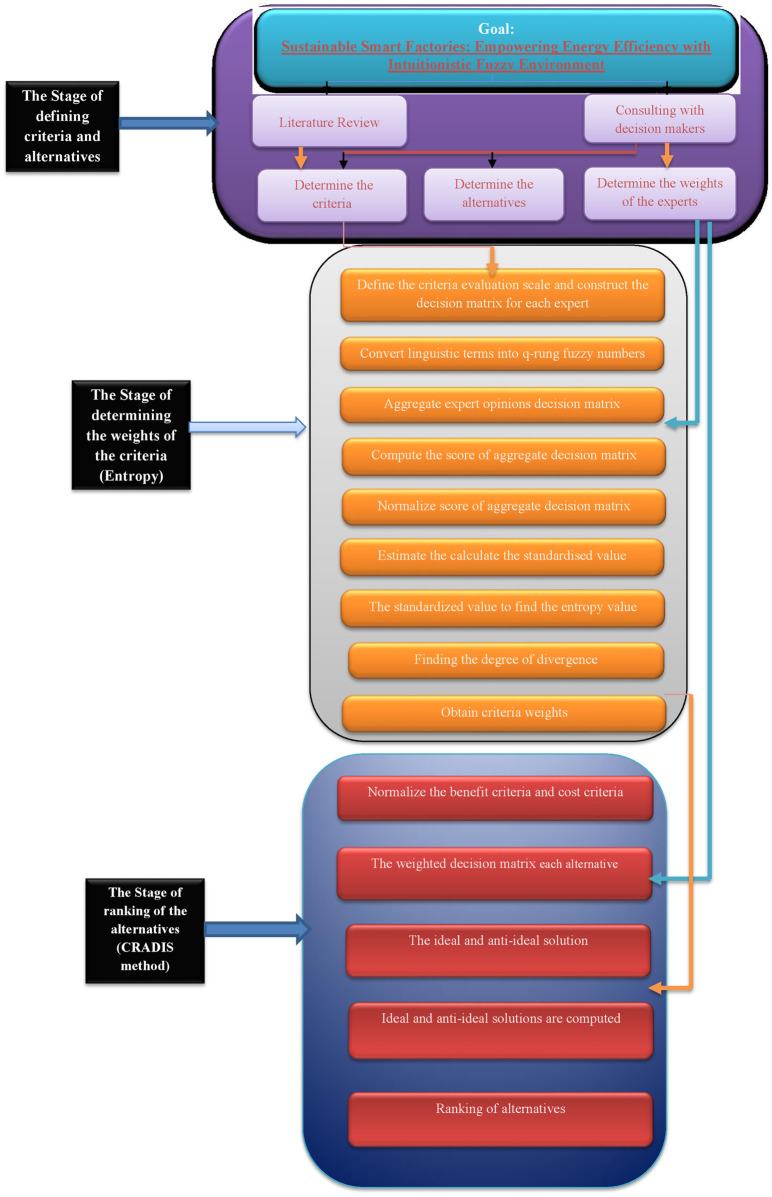
The pictorial view of proposed algorithm.

Puška [56] used the MEREC-CRADIS algorithms, which were improved with double normalization, to improve the selection of electric vehicles. Because of this increase, CRADIS-based judgments in the automobile sector are now more reliable and accurate than in the past. These studies show how versatile and useful CRADIS and its derivatives are in many domains, such as manufacturing, logistics, sustainability, agriculture, economic evaluations, fuzzy environments, occupational risk assessment, and green supplier selection. Table 1 presented a comparative analysis of previous studies, methodologies, key criteria, and contributions, while identifying research gaps in renewable energy. The pictorial view of the proposed algorithm is given in Fig 1.

**Table 1:** Literature review and research gaps.

Author(s)	Year	Methodology	Key Criteria/Focus Areas	Findings/Contributions
Ghenai et al. [57]	2020	SWARA/ARAS hybrid method	Renewable energy indicators	Developed a sustainability indicator model tailored for renewable energy systems.
Chou et al. [58]	2019	Interval-Valued Neutrosophic Sets	Renewable energy selection	Proposed a novel decision-making framework utilizing interval-valued neutrosophic sets.
Dinçer et al. [59]	2023	Hybrid decision-making methodology	Corporate social responsibility, energy industry performance	Established a CSR index for evaluating energy industry performance.
Bilgili et al. [60]	2022	Intuitionistic fuzzy-TOPSIS	Sustainable development, energy alternatives	Evaluated renewable energy alternatives using IFS.
Rane et al. [61]	2023	Various MCDM methods	Sustainable development applications	Reviewed multiple MCDM techniques and their applications in sustainability.
Alkan et al. [62]	2024	CRITIC-SWARA-CODAS	Sustainable energy systems, utilization	Developed a methodology for evaluating renewable energy systems based on sustainability indicators.
Otay et al. [63]	2024	Multi-Expert Pythagorean fuzzy BWM & TOPSIS	Sustainable energy systems, smart cities	Proposed a methodology for evaluating sustainable energy systems in smart cities.
Ali et al. [64]	2023	Various MCDM methods	Hybrid renewable energy resources	Evaluated hybrid renewable energy sources using MCDM techniques.
Almutairi et al. [65]	2022	SWOT analysis, hybrid MCDM, game theory	Optimal renewable energy growth strategies	Developed strategies for renewable energy growth using hybrid MCDM methods.
**Our Study**	**2024**	**IFS, CRADIS, entropy method**	**Energy efficiency, renewable energy use, production efficiency, worker health and safety**	**Introduces a novel framework for evaluating energy efficiency in smart factories, emphasizing real-world applications.**

## 3 Motivation and contribution

### 3.1 Motivation

1. The motivation for this study comes from these two factors: the urgent need to reduce carbon emissions and the rising worry about the environment’s long-term sustainability. To reduce businesses’ influence on the environment, regulations that support energy efficiency must be implemented as quickly as feasible. This is so because the industrial sector is the main source of greenhouse gas emissions.
2. Manufacturers are strongly incentivised to increase energy efficiency as energy expenditures account for a sizeable portion of operating expenses. Reduced operating expenses, improved economic performance, and increased competitiveness are three outcomes that may be attained by optimising energy use.
3. Energy efficiency is becoming much more important to manufacturers to meet strict environmental standards and requirements. It is crucial to have appropriate energy management techniques to prevent fines and reputational harm that might arise from noncompliance with these regulations.
4. The fast progression of intelligent technologies, including automation, AI, and the IoT, has opened up a wide range of prospects for maximising energy efficiency in industrial operations. There have never been more of these chances than there are right now. By implementing these technologies, operational performance and energy efficiency might be greatly improved.

### 3.2 Contributions

- Among the major contributions made by this research are the eight criteria developed and used to assess the energy efficiency of smart factories. It is feasible to examine each of the parts that make up sustainability and energy management using these criteria.
- A strategy utilizing IF environments in combination with MCDM is provided in this research. This framework provides a rational method for prioritizing potential energy efficiency improvement projects based on various factors.
- This paper presents an efficient approach to decision support for energy management. The CRADIS technique is used to rank choices, while the entropy approach is used to provide weight to criteria. It is easier to rank energy efficiency improvement ideas in order of relevance and to assess them objectively when using these approaches.
- Sensitivity analysis is one way to enhance the contribution and assess the dependability and robustness of the findings. This improves the legitimacy of the offered framework and empowers producers to make decisions based on precise facts.
- Beyond its focus on technical details, the article contributes more to the cause of sustainability. A comprehensive strategy for sustainable manufacturing may be achieved with the help of the suggested framework, which links energy efficiency initiatives with ecological and financial goals.

### 3.3 Structure of the paper

An overview of the content of the paper is provided below: Understanding the presented decision-making model requires understanding the fundamental ideas of IFS, which are examined in Section 2. Section illustrates the motivation and contributions. Section 4 details the technique, which includes, among other things, entropy-based criteria weighting and alternative ranking CRADIS. Section 5 illustrates the proposed Algorithm of the IFS based on the Entropy-CRADIS Model. The processes for applying the model and the results of the case study which applies the model to optimizing energy efficiency in smart factories are described in Section 6. This section also presents an overview of the results, an analysis, and recommendations for more environmentally responsible industrial practices studies. Section 7 presents the limitations and discussion of the proposed model. Finally, the conclusion and future directions are presented in Section 8.

## 4 Preliminaries

**Definition 1** [10] *Let* U *be the universal set. A fuzzy set* c in U *is defined as*

$$\begin{array}{c} u=\{\left(c,\omega_{\zeta }\left(c\right)\right):c\in U\}, \end{array}$$

*where ω_ζ_*(*c*) *denotes membership degree of the element t in the universal set U*.

**Definition 2** [9] *An IFS in U is defined as*

$$\begin{array}{c} \chi =\{\langle c,M(c),N(c)|c\in U\rangle \}, \end{array}$$ (1)

*where M*_(*c*)_, *N*_(*c*)_ ∈ [0, 1], such that 0 ≤ *M*_(*c*)_ + *N*_(*c*)_ ≤ 1 *for all c* ∈ *U*. *M*_(*c*)_, *N*_(*c*)_ *represent membership degree and non-membership degree separately for some c* ∈ *X*. *we denote this pair as τ* = (*M*_*τ*_, *N*_*τ*_), *the entirety of this research, and called as IFN satisfying the requirements M*_*τ*_, *N*_*τ*_ ∈ [0, 1] *and M*_*τ*_ + *N*_*τ*_ ≤ 1.

**Definition 3** [66] *When implementing the IFNs in practical life situations, ranking them is necessary. The “score function” (SF) that corresponds to the IFN, τ* = (*M*_*τ*_, *N*_*τ*_) *be defined as*

$$\begin{array}{c} S\left(\tau \right)=M_{\tau }-N_{\tau }. \end{array}$$ (2)

*However, the abovementioned technique is inadequate for categorizing IFNs in several circumstances. For this, an “accuracy function” (AF) H* of *τ is defined as* [67]

$$\begin{array}{c} H\left(\tau \right)=M_{\tau }+N_{\tau }. \end{array}$$ (3)

To enhance this research, provide a fresh scoring method that meets all the criteria mentioned [66].

$$\begin{array}{c} {\overset{˘}{M}}^{\lambda }=\frac{1+M_{\tau }-N_{\tau }}{2}. \end{array}$$ (4)

Next, we’ll take a look at the fundamentals of how to accumulate IFNs.

**Definition 4** [12] Let *τ*_1_ = 〈*M*_1_, *N*_1_〉 and *τ*_2_ = 〈*M*_2_, *N*_2_〉 be two IFNs, λ > 0 then

$$\begin{array}{c} \tau_{1}^{c}=\langle N_{1},M_{1}\rangle \end{array}$$ (5)

$$\begin{array}{c} \tau_{1}\vee \tau_{2}=\langle max\left\{M_{1},M_{2}\right\},min\left\{N_{1},N_{2}\right\}\rangle \end{array}$$ (6)

$$\begin{array}{c} \tau_{1}\wedge \tau_{2}=\langle min\left\{M_{1},M_{2}\right\},max\left\{N_{1},N_{2}\right\}\rangle \end{array}$$ (7)

$$\begin{array}{c} \tau_{1}⊕\tau_{2}=\langle M_{1}+M_{2}-M_{1}M_{2},\;N_{1}N_{2}\rangle \end{array}$$ (8)

$$\begin{array}{c} \tau_{1}⊗\tau_{2}=\langle M_{1}M_{2},\;N_{1}+N_{2}-N_{1}N_{2}\rangle \end{array}$$ (9)

$$\begin{array}{c} \lambda \tau_{1}=\langle 1-(1-M_{{1)}^{\lambda }},\;N_{1}^{\lambda }\rangle \end{array}$$ (10)

$$\begin{array}{c} \tau_{1}^{\lambda }=\langle M_{1}^{\lambda },\;1-{\left(1-N_{1}\right)}^{\lambda }\rangle \end{array}$$ (11)

### 4.1 Intuitionistic fuzzy Dombi operator

**Theorem 1** [68] *Let* ${\tilde{\beta }}_{i}=\left(M_{i},N_{i}\right)$ (*i* = 1, 2, …, *n*) *be a set of IFNs. Then, the intuitionistic fuzzy Dombi weighted average (IFDWA) operator is a function β*^*n*^ → *β*, *such that*:

$$\begin{array}{c} \text{IFDWA}\left(\beta_{1},\beta_{2},\ldots ,\beta_{n}\right)=\overset{n}{\underset{i=1}{⊗}}{\left(\beta_{i}\right)}^{{\eta^{\gamma }}_{i}}, \end{array}$$ (12)

*where η*^*γ*^ = (*η*^*γ*^_1_, *η*^*γ*^_2_, …, *η*^*γ*^_*n*_)^*t*^ *is the weight vector of β*_*i*_ (*i* = 1, 2, …, *n*), *η*^*γ*^_*i*_ > 0, *and* $\sum_{i=1}^{n}{\eta^{\gamma }}_{i}=1$.

**Theorem 2** *Let* ${\tilde{\beta }}_{i}=\left(M_{i},N_{i}\right)$ (*i* = 1, 2, …, *n*) *be a set of IFNs. Then, the aggregated value of them using the IFDWA operation is also an IFN and is given by*:

$$\begin{array}{c} \begin{array}{cc} & \text{IFDWA}\left({\tilde{\beta }}_{1},{\tilde{\beta }}_{2},\ldots ,{\tilde{\beta }}_{n}\right)=\overset{n}{\underset{i=1}{⊕}}{\eta^{\gamma }}_{i}{\tilde{\beta }}_{i}\\ & =(1-\frac{1}{1+{\left\{\sum_{i=1}^{n}{\eta^{\gamma }}_{i}{\left(\frac{M_{i}}{1-M_{i}}\right)}^{m}\right\}}^{\frac{1}{m}}},\frac{1}{1+{\left\{\sum_{i=1}^{n}{\eta^{\gamma }}_{i}{\left(\frac{1-N_{i}}{N_{i}}\right)}^{m}\right\}}^{\frac{1}{m}}}) \end{array} \end{array}$$ (13)

*where η*^*γ*^ = (*η*^*γ*^_1_, *η*^*γ*^_2_, …, *η*^*γ*^_*n*_)^*t*^ *is the weight vector of* ${\tilde{\beta }}_{i}$ (*i* = 1, 2, …, *n*), *η*^*γ*^_*i*_ > 0, *and* $\sum_{i=1}^{n}{\eta^{\gamma }}_{i}=1$.

**Theorem 3** [68] *Let* ${\tilde{\beta }}_{i}=\left(M_{i},N_{i}\right)$ (*i* = 1, 2, …, *n*) *be a set of IFNs. Then, the Intuitionistic Fuzzy Dombi Weighted Geometric (IFDWG) operator is a function* ${\tilde{\beta }}^{n}\to \tilde{\beta }$, *such that*:

$$\begin{array}{c} \text{IFDWG}\left({\tilde{\beta }}_{1},{\tilde{\beta }}_{2},\ldots ,{\tilde{\beta }}_{n}\right)=\overset{n}{\underset{i=1}{⊗}}{\left({\tilde{\beta }}_{i}\right)}^{{{\eta_{i}}^{\gamma }}_{i}}, \end{array}$$ (14)

*where η*^*γ*^ = (*η*^*γ*^_1_, *η*^*γ*^_2_, …, *η*^*γ*^_*n*_)^*t*^ *is the weight vector of* ${\tilde{\beta }}_{i}$ (*i* = 1, 2, …, *n*), *η*^*γ*^_*i*_ > 0, *and* $\sum_{i=1}^{n}{\eta^{\gamma }}_{i}=1$.

**Theorem 4** *Let* ${\tilde{\beta }}_{i}=\left(M_{i},N_{i}\right)$ (*i* = 1, 2, …, *n*) *be a set of IFNs. Then, the aggregated value of them using the IFDWG operation is also an IFN and is given by*:

$$\begin{array}{c} \begin{array}{cc} & \text{IFDWG}({\tilde{\beta }}_{1},{\tilde{\beta }}_{2},\ldots ,{\tilde{\beta }}_{n})\\ & =\overset{n}{\underset{i=1}{⊗}}{\left({\tilde{\beta }}_{i}\right)}^{{\eta^{\gamma }}_{i}}\\ & =(\frac{1}{1+{\left\{\sum_{i=1}^{n}{\eta^{\gamma }}_{i}{\left(\frac{1-M_{i}}{M_{i}}\right)}^{m}\right\}}^{\frac{1}{m}}},1-\frac{1}{1+{\left\{\sum_{i=1}^{n}{\eta^{\gamma }}_{i}{\left(\frac{N_{i}}{1-N_{i}}\right)}^{m}\right\}}^{\frac{1}{m}}}) \end{array} \end{array}$$ (15)

*where M* = (*η*^*γ*^_1_, *η*^*γ*^_2_, …, *η*^*γ*^_*n*_)^*t*^ is the weight vector of ${\tilde{\beta }}_{i}$ (*i* = 1, 2, …, *n*), *η*^*γ*^_*i*_ > 0, *and* $\sum_{i=1}^{n}{\eta^{\gamma }}_{i}=1$.

## 5 Algorithm of the IFS based on Entropy-CRADIS Model

A decision-making problem consists of a set of criteria, represented as *Cr*_*j*_ = *Cr*_1_, *Cr*_2_, …, *Cr*_*u*_, and a set of alternatives, represented as *G*_*i*_ is equal to *G*_1_, *G*_2_, *G*_*r*_. Many decision-makers (DMs) utilize IFNs to express their opinions and ideas on the alternatives *G*_*i*_ based on each criterion *Cr*_*i*_. M = [*Cr*_*ij*_]_*u*×*v*_ represents the IF decision maker (DM) matrix, which is a representation of the assessment of given options under several criteria, as reported by k experts.


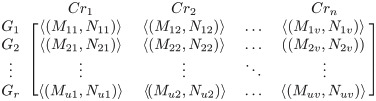



As *Cr*_*ij*_ = 〈(*M*_*ij*_, *N*_*ij*_)〉 for *i* = 1, …, *u* and *j* = 1, …, *v*, Table 2 defines the eight IF linguistic words that decision makers (DMs) use

**Table 2:** Linguistic term.

Linguistic term	Abbreviation	IFNs
extremely good	AA	〈0.93, 0.05〉
very good	AB	〈0.90, 0.7〉
good	AC	〈0.85, 0.13〉
slightly good	AD	〈0.80, 0.15〉
fair	AE	〈0.70, 0.25〉
slightly poor	AF	〈0.65, 0.30〉
poor	AP	〈0.50, 0.45〉
Very poor	AJ	〈0.40, 0.55〉

**Step 1:** Using the score function provided by Eq (2) to assign values to each decision maker, determine the weights of the decision makers listed in Table 3. This is accomplished by considering the different responsibilities and functions that each decision-maker has while making decisions. Enter these score values to finish the calculation that is presented below.
  $$\begin{array}{c} K_{t}=\frac{\sum_{t=1}^{k}\left(S_{\left(\mu_{t}\right)}\right)}{\sum_{q=1}^{l}\left[\sum_{t=1}^{k}\left(S_{\left(\mu_{t}\right)}\right)\right]}\;t=1,2,\ldots ,k \end{array}$$ (16)
**Step 2:** To obtain the aggregated IFNs, each table must be gathered into a single group. This aims to obtain the combined IFNs Table 3 based on expert opinions. More specifically, it is made feasible by the IFDWA operator using Eq 17.
  $$\begin{array}{c} \begin{array}{cc} & \text{IFDWA}\left({\tilde{\beta }}_{1},{\tilde{\beta }}_{2},\ldots ,{\tilde{\beta }}_{n}\right)=\overset{n}{\underset{i=1}{⊕}}{\eta^{\gamma }}_{i}{\tilde{\beta }}_{i}\\ & =(1-\frac{1}{1+{\left\{\sum_{i=1}^{n}{\eta^{\gamma }}_{i}{\left(\frac{M_{i}}{1-M_{i}}\right)}^{m}\right\}}^{\frac{1}{m}}},\frac{1}{1+{\left\{\sum_{i=1}^{n}{\eta^{\gamma }}_{i}{\left(\frac{1-N_{i}}{N_{i}}\right)}^{m}\right\}}^{\frac{1}{m}}}) \end{array} \end{array}$$ (17)
**Step 3:** The next step in the procedure is to determine the aggregated score matrix. One method to accomplish this is using the supplied formula to calculate the score value of each element in the aggregated matrix.
  $$\begin{array}{c} S\left(\tau \right)=M_{\tau }-N_{\tau } \end{array}$$

**Table 3:** Experts information for evaluation.

Decision Maker	Role and Responsibility	Experience and Qualification
Production Manager	Oversees production processes, ensuring efficiency and quality. Responsible for implementing production strategies and optimizing efficiency.	Bachelor’s degree in mechanical engineering and 10 years of experience in manufacturing
Sustainability Officer	Develops and implements sustainability initiatives and strategies. Manages sustainability projects and ensures compliance with environmental regulations	Master’s degree in environmental science and 8 years of experience in sustainable practices
Chief Technology Officer	Leads technology integration and innovation efforts in the factory. Implements advanced technology solutions to optimize energy use and enhance operational efficiency.	PhD in Computer Science and 15 years of experience in IoT and AI technologies

### 5.1 Entropy method

Using the entropy technique, the weights of the criterion were determined.

**Step 4:** Normalisation is a process of converting the input data of a decision-making matrix into a range of values between 0 and 1. Normalization is a crucial technique for streamlining the process when the criteria are given a range of numerical values. Eqs (18) and(24) were used to normalize the data using the entropy approach for the benefit type and cost type criteria.
  $$\begin{array}{c} \nu_{ij}=\frac{k_{ij}-\underset{i}{min}\left(k_{ij}\right)}{\underset{i}{max}\left(k_{ij}\right)-\underset{i}{min}\left(k_{ij}\right)}\;i=1,2,\ldots ,u;\;j=1,2,\ldots ,v; \end{array}$$ (18)
  and
  $$\begin{array}{c} \nu_{ij}=\frac{\underset{i}{max}\left(k_{ij}\right)-k_{ij}}{\underset{i}{max}\left(k_{ij}\right)-\underset{i}{min}\left(k_{ij}\right)}\;i=1,2,\ldots ,u;\;j=1,2,\ldots ,v \end{array}$$ (19)
**Step 5:** This stage involves computing the standardized value, denoted by the notation *S*_*ij*_ using Eq 20.
  $$\begin{array}{c} S_{ij}=\frac{\nu_{ij}}{\sum_{i=1}^{m}\left(\nu_{ij}\right)}\;i=1,2,\ldots ,u;\;j=1,2,\ldots ,v; \end{array}$$ (20)
**Step 6:** Eq 21 illustrates how the standardized value is utilized to calculate the entropy value of the *jth* criteria.
  $$\begin{array}{c} \nu_{j}=P\sum_{i=1}^{m}\left(S_{ij}ln\left(S_{ij}\right)\right)\;j=1,2,\ldots ,v; \end{array}$$ (21)
  Where,
  $$\begin{array}{c} P=\frac{1}{ln(r)} \end{array}$$
**Step 7:** Finding the degree of divergence *Y*_*j*_ for each of the listed criteria is the first step towards figuring out the weights of the jth criterion.
  $$\begin{array}{c} Y_{j}=1-\nu_{j}\;j=1,2,\ldots ,v; \end{array}$$

Based on divergence measure weights are calculated as,

$$\begin{array}{c} N_{j}=\frac{Y_{j}}{\sum_{j=1}^{n}\left(Y_{j}\right)}\;j=1,2,\ldots ,v; \end{array}$$ (22)

such that, *N*_*i*_ ∈ [0, 1] and $\sum_{i=1}^{n}N_{i}=1$.

The weight becomes excessive when *S*_*ij*_ = 0 and we compute *ν*_*j*_ by imposing the restriction *S*_*ij*_ *ln*(*S*_*ij*_) = 0. This is because the entropy value decreases for such values [69]. This is due to the high number of zeros in the measured data set *S*_*ij*_. To avoid having zero values in the normalized data set, the standardization process provided in Eq 20 is modified and stated as Eq (23).

$$\begin{array}{c} S_{ij}=\frac{\nu_{ij}+k}{\sum_{i=1}^{m}\left(\nu_{ij}+k\right)}\;i=1,2,\ldots ,u;\;j=1,2,\ldots ,v \end{array}$$ (23)

Where k is a constant satisfying the condition,

$$\begin{array}{c} \nu_{ij}+k>0. \end{array}$$

### 5.2 CRADIS method

To utilize the IFS CRADIS method, take the following actions:

**Step 8:** Create a normalised matrix $N^{\prime }={\left(R_{ij}^{*}\right)}_{u\times v}$ from the scoring matrix M^*^ as follows using Eq 24:
  $$\begin{array}{c} R_{ij}^{*}=\{\begin{array}{c} \frac{R_{ij}}{R_{j\;max}},\;\text{if}\;j\in E_{b},\\ \frac{R_{j\;min}}{R_{ij}},\;\text{if}\;j\in F_{b}, \end{array}\;i=1,2,\ldots ,u;\;j=1,2,\ldots ,v \end{array}$$ (24)
  where *E*_*b*_ indicates the benefit type of criteria and *F*_*b*_ indicates the cost type criteria.
**Step 9:** Eq 25 forms the weighted decision matrix *X* = (*x*_*ij*_)_*u*×*v*_:
  $$\begin{array}{c} x_{ij}=R_{ij}^{*}w_{j}\;i=1,2,\ldots ,u;\;j=1,2,\ldots ,v \end{array}$$ (25)
**Step 10:** The following Eqs 26 and 27 are used to get the ideal and anti-ideal solution::
  $$\begin{array}{c} l_{i}=max\;x_{ij}\;i=1,2,\ldots ,u; \end{array}$$ (26)
  $$\begin{array}{c} l_{ai}=min\;x_{ij}\;i=1,2,\ldots ,u; \end{array}$$ (27)
**Step 11:** Eqs 28 and 29 are used to determine deviations from ideal and anti-ideal solutions.
  $$\begin{array}{c} q_{ij}^{+}=l_{i}-x_{ij} \end{array}$$ (28)
  $$\begin{array}{c} q_{ij}^{-}=x_{ij}-l_{ai} \end{array}$$ (29)
**Step 12:** Eqs 30 and 31 may be used to calculate the deviation degrees of each alternative from the ideal and anti-ideal solutions, respectively.
  $$\begin{array}{c} p_{i}^{+}=\sum_{j=1}^{n}q_{ij}^{+}\;i=1,2,\ldots ,u; \end{array}$$ (30)
  $$\begin{array}{c} p_{i}^{-}=\sum_{j=1}^{n}q_{ij}^{-}\;i=1,2,\ldots ,u; \end{array}$$ (31)
**Step 13:** The calculation of the utility function for each option with respect to how much it deviates from the best options.
  $$\begin{array}{c} O_{i}^{+}=\frac{p_{0}^{+}}{p_{i}^{+}}\;i=1,2,\ldots ,u; \end{array}$$ (32)
  $$\begin{array}{c} O_{i}^{-}=\frac{s_{i}^{-}}{s_{0}^{-}}\;i=1,2,\ldots ,u; \end{array}$$ (33)
  where $p_{0}^{+}=minp_{i}^{+}$ and $p_{0}^{-}=maxp_{i}^{-}$.
**Step 14:** Eq 34 calculated the degree of value to determine the final ranking.
  $$\begin{array}{c} R_{i}={\left(O_{i}^{+}\right)}^{P^{\Omega }}+{\left(O_{i}^{-}\right)}^{\left(1-P^{\Omega }\right)}\;i=1,2,\ldots ,u \end{array}$$ (34)

## 6 Applications of the proposed framework

The study’s major focus is optimizing energy efficiency in smart manufacturing. This results from the growing demand for companies to lower their operational costs and carbon footprint to abide by strict environmental requirements. Measures to improve energy efficiency are therefore becoming more and more important. Energy efficiency may be maximized in smart factories, which are made feasible by cutting-edge technologies like AI and the IoT. However, analyzing and choosing the best tactics to increase energy efficiency usually presents difficulties for decision-makers. Because of this, the goal of this paper is to present a thorough framework for decision-making in IF environments by using MCDM. The framework will utilize the entropy method for criterion weighting, the CRADIS method for alternative ranking, and sensitivity analysis to evaluate the robustness of the results to identify key criteria, including energy consumption, operational costs, carbon emissions, resource utilization efficiency, technology integration, production efficiency, and employee health and safety. The robustness of the findings will be assessed using these techniques. The framework that has been made available will help manufacturers overcome these obstacles and decide intelligently how to increase energy efficiency in the operations of smart factories. This will support the promotion of environmentally friendly production methods.

### 6.1 Definition of alternatives

1. **Advanced Energy Management Systems (AEMS) (*G*_1_):** With the use of cutting-edge technology, energy usage can be tracked, managed, and improved in real-time. Using these systems, you can save substantial money and energy since they show you how much energy you use, where the inefficiencies are, and how to automate your energy savings.
2. **Renewable Energy Integration (*G*_2_):** The factory is powered by renewable energy sources, including biomass, solar energy, and wind turbines. This strategy may save money in the long run since it lessens the strain on non-renewable power sources and lowers carbon emissions. It uses sustainable energy sources to achieve all of these goals.
3. **Energy-Efficient Equipment and Technologies (*G*_3_):** the application of energy-saving technologies, such as LED lighting and high-efficiency equipment. These technologies not only help to reduce operating expenses and energy consumption, but they also help to lessen the negative environmental effects. Examples include modern heating, ventilation, air conditioning systems, high-efficiency motors, and variable frequency drives.
4. **Process Optimization and Automation (*G*_4_):** Automating routine tasks and industrial processes can help eliminate inefficiencies and save energy. The aforementioned approach enhances productivity, minimizes mistakes, and optimizes energy consumption. It is based on technologies like robotic process automation, lean manufacturing, and predictive maintenance.
5. **Employee Training and Engagement Programs (*G*_5_):** A component of the endeavor to promote an ecologically conscious culture includes informing and training staff members on ways to reduce their energy use. Giving workers more autonomy in reducing energy usage is an easy way to increase their satisfaction and productivity. Some instances of effective projects are as follows: ongoing communication on sustainable goals, seminars, and incentive schemes.

### 6.2 Definition of criteria

1. **Energy Consumption: (*Cr*_1_):** The whole kilowatt-hour (kWh) consumption of the manufacturing facility for internal activities. An organization may evaluate its efficiency using less energy as it directly affects operating costs and carbon emissions. Monitoring energy usage can yield real-time data that can be used for optimization, thanks to the use of smart meters and management systems.
2. **Operational Cost:(*Cr*_2_):** This sum represents the whole cost of all energy-related expenses, such as gasoline, electricity, and maintenance. Reducing spending on these expenses might lead to a notable rise in income. Effective energy management facilitates the process of identifying cost-saving and economic performance-boosting strategies. These two objectives might be completed at the same time.
3. **Carbon Emissions: (*Cr*_3_):** The quantity of greenhouse gases emitted during the energy-related processes is measured in CO2 equivalents. There is no greater need for environmental protection and regulatory compliance than reducing carbon emissions. It can significantly decrease emissions by utilizing renewable energy sources and energy-efficient activities.
4. **Resource Utilization Efficiency (*Cr*_4_):** The degree to which manufacturing processes utilize available resources effectively is known as manufacturing efficiency. Economical use reduces waste, drives costs, and promotes eco-friendly production practices. A comparison of the input and output materials and an analysis of waste management practices may be used to assess this.
5. **Technology Integration (*Cr*_5_):** The effectiveness of cutting-edge technology in reducing energy usage and its integration, including automation, AI, and IoT are also significant factors. These technologies can improve energy management and operational efficiency when strongly integrated. They accomplish this by offering thorough data and the capacity for forecasting.
6. **Renewable Energy Utilization (*Cr*_6_):** The majority of the plant’s energy comes from sustainable resources, including biomass, solar, and wind energy. It may be possible to increase the use of renewable energy sources, decreasing both the carbon footprint and reliance on fossil fuels. One measure that may be used to assess the advancements in this area is the proportion of energy produced from renewable sources.
7. **Production Efficiency (*Cr*_7_):** The efficiency of a manufacturing process may be determined by dividing its energy input by its final product. Less waste is produced by more efficient energy usage, which lowers costs and lessens environmental harm. By introducing new technologies and modernizing current practices, this metric might be made even better.
8. **Employee Health and Safety (*Cr*_8_):** The impact that energy-saving initiatives have on employees’ health and safety, including their working environments and the risks to which they are now exposed. Providing a safe working environment for employees is crucial due to the clear relationship between their happiness, well-being, and productivity. To monitor this criterion, you may poll your staff and go through health and safety information.

### 6.3 Experimental results

Table 2 has a set of alternatives and criteria. Table 4 shows that utilizing IFNs, several DMs provide their opinions on each of the criteria *Cr*_*i*_ for the alternatives *G*_*i*_.

**Table 4:** Evaluations of each alternative.

DMs	Alternative	*C*_1_	*C*_2_	*C*_3_	*C*_4_	*C*_5_	*C*_6_	*C*_7_	*C*_8_
*DM*_1_	*G*_1_	AA	AB	AC	AD	AE	AF	AP	AF
	*G*_2_	AJ	AB	AC	AJ	AF	AE	AP	AF
	*G*_3_	AF	AP	AJ	AC	AA	AE	AC	AD
	*G*_4_	AE	AB	AP	AE	AF	AJ	AC	AF
	*G*_5_	AC	AF	AB	AD	AD	AA	AJ	AD
*DM*_2_	*G*_1_	AP	AE	AF	AA	AJ	AC	AB	AD
	*G*_2_	AB	AF	AP	AC	AJ	AE	AA	AC
	*G*_3_	AC	AJ	AB	AF	AP	AD	AE	AD
	*G*_4_	AE	AF	AC	AJ	AA	AP	AB	AD
	*G*_5_	AF	AA	AD	AP	AJ	AC	AB	AE
*DM*_3_	*G*_1_	AF	AP	AC	AE	AB	AA	AJ	AP
	*G*_2_	AB	AF	AE	AP	AJ	AC	AP	AD
	*G*_3_	AC	AP	AD	AJ	AB	AE	AF	AJ
	*G*_4_	AE	AJ	AB	AF	AC	AA	AP	AC
	*G*_5_	AP	AB	AE	AC	AD	AJ	AF	AD

**Step 1:** Use Eq 16 to get the decision makers’ weights. Table 5 displays the DM weights.
**Step 2:** Eq 17 may be used to produce the combined IFNs Table based on the opinions of the DMs. Please refer to Table 6 for compiling all the findings.
**Step 3:** Use 2 to find the aggregated score matrix. What follows is a matrix with the score values of each element.
  $$Sc_{ij}=[\begin{array}{cccccccc} 0.8739 & 0.8400 & 0.7041 & 0.5042 & 0.5095 & 0.5517 & 0.4379 & 0.2942\\ 0.7675 & 0.7919 & 0.6777 & 0.5011 & 0.2822 & 0.4913 & 0.3969 & 0.0900\\ 0.8683 & 0.8192 & 0.5635 & 0.6059 & 0.5080 & 0.3541 & 0.2653 & 0.0554\\ 0.7542 & 0.6969 & 0.5555 & 0.6971 & 0.4464 & 0.5111 & 0.3407 & 0.0963\\ 0.7486 & 0.8951 & 0.5524 & 0.5338 & 0.5326 & 0.3545 & 0.2756 & -0.0940 \end{array}]$$

**Table 5:** Decision makers weights.

DM	Decision Maker’s Profession	Role and responsibility	Experience	Weights
DM1	〈0.95, 0.03〉	〈0.85, 0.10〉	〈0.80, 0.15〉	0.4034
DM2	〈0.90, 0.05〉	〈0.80, 0.15〉	〈0.70, 0.25〉	0.3130
DM3	〈0.85, 0.10〉	〈0.70, 0.25〉	〈0.40, 0.55〉	0.2836

**Table 6:** Aggregated decision matrix.

*Cr*_*i*_	*G*_1_	*G*_2_	*G*_3_	*G*_4_	*G*_5_
*Cr*_1_	〈0.9302,0.0563〉	〈0.8329,0.0654〉	〈0.9225,0.0542〉	〈0.8565,0.1023〉	〈0.8214,0.0728〉
*Cr*_2_	〈0.9032,0.0632〉	〈0.8704,0.0785〉	〈0.9034,0.0842〉	〈0.8324,0.1355〉	〈0.9382,0.0431〉
*Cr*_3_	〈0.8342,0.1301〉	〈0.8250,0.1473〉	〈0.7643,0.2008〉	〈0.7387,0.1832〉	〈0.7539,0.2015〉
*Cr*_4_	〈0.7347,0.2305〉	〈0.7543,0.2532〉	〈0.7034,0.0975〉	〈0.8045,0.1074〉	〈0.7352,0.2014〉
*Cr*_5_	〈0.7233,0.2138〉	〈0.6432,0.3610〉	〈0.7382,0.2302〉	〈0.70240,0.2560〉	〈0.7064,0.1738〉
*Cr*_6_	〈0.7554,0.2037〉	〈0.7496,0.2583〉	〈0.6277,0.2736〉	〈0.7274,0.2163〉	〈0.6076,0.2531〉
*Cr*_7_	〈0.6532,0.2153〉	〈0.6432,0.2463〉	〈0.6232,0.3579〉	〈0.6528,0.3121〉	〈0.6023,0.3267〉
*Cr*_8_	〈0.6154,0.3212〉	〈0.5432,0.4532〉	〈0.5092,0.4538〉	〈0.5323,0.436〉	〈0.43803,0.5320〉

### 6.4 Entropy method for criteria weights

**Step 4:** Data normalisation is the process of transforming the input data of a decision-making matrix into a binary range of zeros and ones. Eq 18 was used for benefit type criteria in EM, whereas Eq 19 was used for cost type criteria.
  $$\nu_{ij}=[\begin{array}{cccccccc} 1.0000 & 0.7220 & 1.0000 & 0.0158 & 0.9077 & 0.0000 & 0.0000 & 1.0000\\ 0.1508 & 0.4793 & 0.8260 & 0.0000 & 0.0000 & 0.3057 & 0.2375 & 0.4740\\ 0.9553 & 0.6171 & 0.0732 & 0.5347 & 0.9018 & 1.0000 & 1.0000 & 0.3849\\ 0.0447 & 0.0000 & 0.0204 & 1.0000 & 0.6558 & 0.2055 & 0.5632 & 0.4902\\ 0.0000 & 1.0000 & 0.0000 & 0.1668 & 1.0000 & 0.9980 & 0.9403 & 0.0000 \end{array}]$$
**Step 5:** Using Eq 23 and k = 0.5, the standardised value *S*_*ij*_ is computed during this phase.
  $$SD_{ij}=[\begin{array}{cccccccc} 0.3225 & 0.2298 & 0.3394 & 0.1223 & 0.2360 & 0.0998 & 0.0954 & 0.3093\\ 0.1399 & 0.1841 & 0.3000 & 0.1186 & 0.0838 & 0.1608 & 0.1407 & 0.2009\\ 0.3129 & 0.2100 & 0.1297 & 0.2453 & 0.2350 & 0.2995 & 0.2862 & 0.1825\\ 0.1171 & 0.0940 & 0.1178 & 0.3557 & 0.1937 & 0.1408 & 0.2029 & 0.2042\\ 0.1075 & 0.2820 & 0.1131 & 0.1581 & 0.2515 & 0.2991 & 0.2748 & 0.1031 \end{array}]$$
**Step 6:** To find the entropy value of the *jth* criterion, we use Eq 21.
  $$M_{j}=[\begin{array}{cccccccc} 0.9287 & 0.9671 & 0.9266 & 0.9406 & 0.9655 & 0.9457 & 0.9548 & 0.9658 \end{array}]$$
**Step 7:** Eq 22 calculates the weights of the *jth* condition.
  $$N_{j}=[\begin{array}{cccccccc} 0.1761 & 0.0812 & 0.1812 & 0.1466 & 0.0851 & 0.1340 & 0.1115 & 0.0843 \end{array}]$$

### 6.5 CRADIS method

The following is a rundown of the CRADIS procedure.

**Step 8:** The scoring matrix M^*^ is used to create the normalised decision matrix *N*^′^, which is then expressed as:
  $$\begin{array}{c} N^{\prime }=[\begin{array}{cccccccc} 1.0000 & 0.8296 & 1.0000 & 0.7233 & 0.9566 & 1.0000 & 1.0000 & 1.0000\\ 0.8782 & 0.8800 & 0.9625 & 0.7188 & 0.5299 & 0.8905 & 0.9064 & 0.3059\\ 0.9936 & 0.8507 & 0.8003 & 0.8692 & 0.9538 & 0.6418 & 0.6058 & 0.1883\\ 0.8630 & 1.0000 & 0.7890 & 1.0000 & 0.8382 & 0.9264 & 0.7780 & 0.3273\\ 0.8566 & 0.7786 & 0.7845 & 0.7657 & 1.0000 & 0.6426 & 0.6294 & -0.3195 \end{array}] \end{array}$$
**Step 9:** We construct the weighted decision matrix D by first utilising the goal weights, which are determined by the entropy, and then by applying Eq 25.
  $$\begin{array}{c} D=[\begin{array}{cccccccc} 0.1761 & 0.0674 & 0.1812 & 0.1060 & 0.0814 & 0.1340 & 0.1115 & 0.0843\\ 0.1546 & 0.0714 & 0.1744 & 0.1054 & 0.0451 & 0.1193 & 0.1011 & 0.0258\\ 0.1749 & 0.0691 & 0.1450 & 0.1274 & 0.0811 & 0.0860 & 0.0676 & 0.0159\\ 0.1520 & 0.0812 & 0.1430 & 0.1466 & 0.0713 & 0.1241 & 0.0867 & 0.0276\\ 0.1508 & 0.0632 & 0.1422 & 0.1123 & 0.0851 & 0.0861 & 0.0702 & -0.0269 \end{array}] \end{array}$$
**Step 10:** Table 7 displays the outcomes of utilising Eqs 26 and 27, respectively, to ascertain the ideal and anti-ideal solutions.
**Step 11:** The calculated deviations from ideal solutions, *q*^+^, are displayed in Eq 35 and are obtained from Eq 28.
  $$\begin{array}{c} D^{+}=(\begin{array}{ccccc} 0.6749 & 0 & 0.7463 & 0.5156 & 0.4993\\ 0.0489 & 0.3650 & 0.1201 & 0 & 0.2388\\ 0.3094 & 0 & 0.4597 & 0.1189 & 0.3024\\ 0.0826 & 0.3378 & 0.1000 & 0.0436 & 0\\ 0.1098 & 0.0092 & 0 & 0.1537 & 0.1973 \end{array}) \end{array}$$ (35)
  *q*^−^ represents the deviations from anti-ideal solutions, which are calculated using Eq 29 and shown in Eq 36.
  $$\begin{array}{c} D^{-}=[\begin{array}{cccccccc} 0.0051 & 0.1139 & 0.0000 & 0.0752 & 0.0999 & 0.0472 & 0.0697 & 0.0969\\ 0.0198 & 0.1030 & 0.0000 & 0.0690 & 0.1294 & 0.0551 & 0.0734 & 0.1486\\ 0.0000 & 0.1059 & 0.0299 & 0.0475 & 0.0938 & 0.0889 & 0.1074 & 0.1591\\ 0.0000 & 0.0708 & 0.0090 & 0.0053 & 0.0807 & 0.0278 & 0.0652 & 0.1244\\ 0.0000 & 0.0876 & 0.0087 & 0.0386 & 0.0658 & 0.0647 & 0.0807 & 0.1778 \end{array}] \end{array}$$ (36)
**Step 12:** Eqs 30 and 31 are used to derive the deviation degrees of each option from the ideal and anti-ideal solutions. The findings are shown in Table 8.
**Step 13:** Find the alternatives’ utility functions. According to Table 9, the utility functions $O_{i}^{+}$ and $O_{i}^{-}$ under the largest and smallest deviation, respectively, are calculated using Eqs 32 and 33, depending on the degree of deviation.
**Step 14:** Table 10 displays the results of solving Eq 34 with *P*^Ω^ = 0.5.

**Table 7:** Ideal and anti-ideal solutions.

Ideal solution	Anti-ideal solution
*l*_*i*_	*l*_*ai*_
0.1812	0.0674
0.1744	0.0258
0.1749	0.0159
0.1520	0.0276
0.1508	-0.0269

**Table 8:** Deviation degrees.

Alternatives	$P_{i}^{+}$	$P_{i}^{-}$
*G*_1_	0.0674	0.4031
*G*_2_	0.0258	0.5908
*G*_3_	0.0159	0.6400
*G*_4_	0.0276	0.6117
*G*_5_	-0.0269	0.8984

**Table 9:** Utility function of alternatives.

Alternatives	$O_{i}^{+}$	$O_{i}^{-}$
*G*_1_	0.7544	0.4486
*G*_2_	0.6404	0.6575
*G*_3_	0.6057	0.7123
*G*_4_	1.0000	0.6808
*G*_5_	0.7315	1.0000

**Table 10:** Average deviation.

Alternatives	*R*_*i*_	Ranking
*G*_1_	1.5384	5
*G*_2_	1.6112	4
*G*_3_	1.6223	3
*G*_4_	1.8251	2
*G*_5_	1.8553	1

Hence, *G*_5_ is the best choice.

### 6.6 Sensitivity analysis

**Fig 2:**
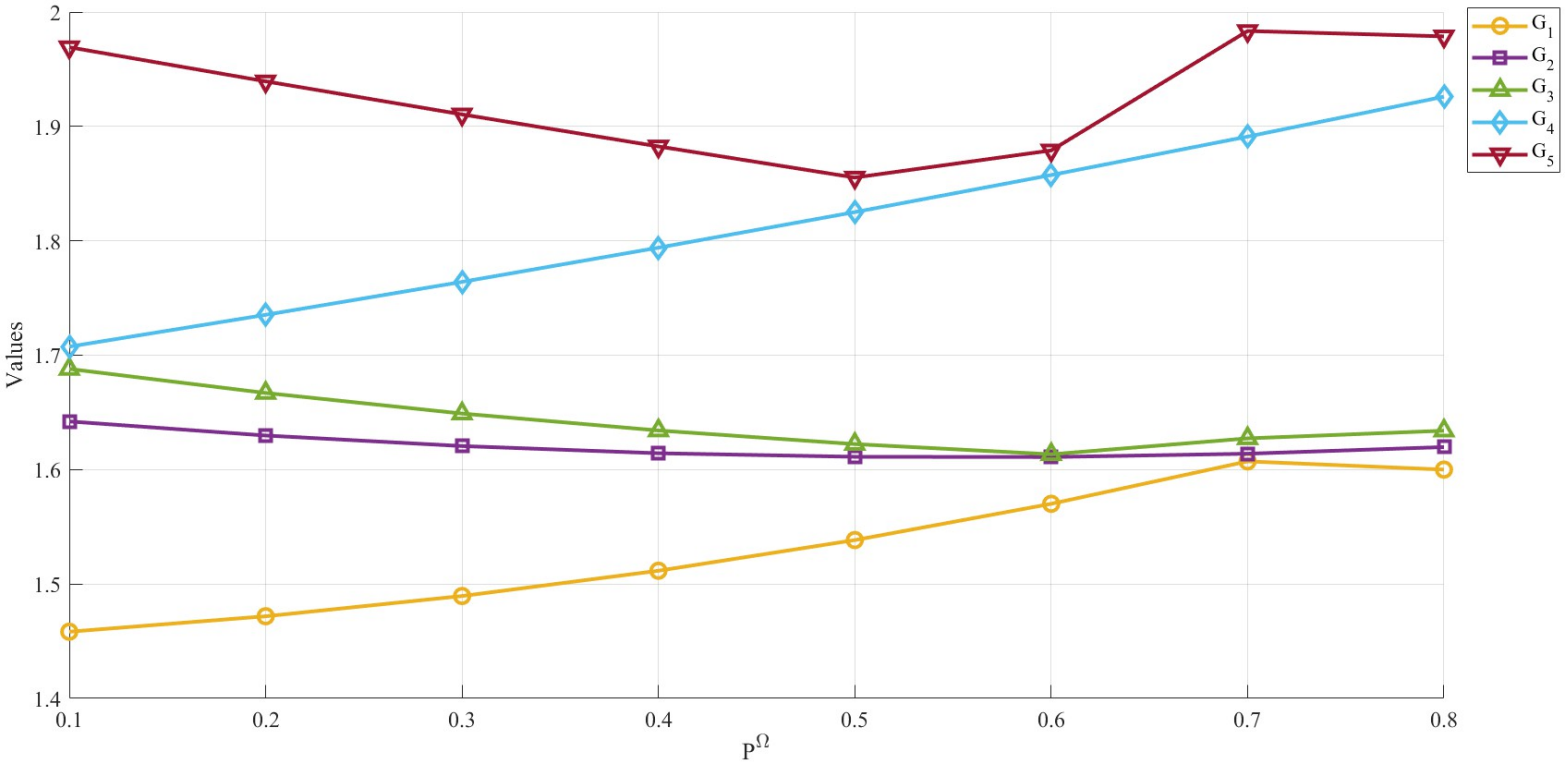
Variations with parameter (*P*^Ω^).

When evaluating the decision-making process’s robustness and dependability, sensitivity analysis has shown to be a huge help. Sensitivity analysis involves methodically changing the model’s input parameters or assumptions to see how different values affect the ranks of the outcomes. By conducting sensitivity analyses, faculty recruitment decision-makers can learn how the model reacts to changes in criterion weights, data inputs, or decision thresholds regarding application ranks, as well as which variables impact application ranks most. The use of sensitivity analysis helps decision-makers make clearer and more solid choices. In Fig 2 and Table 11, we can see how different *P*^Ω^ values affect the decision-making inside the framework.

**Table 11:** The influence of the parameter *P*^Ω^ on the outcome of the decision.

*P* ^Ω^	*G*_1_	*G*_2_	*G*_3_	*G*_4_	*G*_5_	Ranking
*P*^Ω^ = 0.1	1.4583	1.6421	1.6880	1.7075	1.9692	*G*_5_ ≻ *G*_4_ ≻ *G*_3_ ≻ *G*_2_ ≻ *G*_1_
*P*^Ω^ = 0.2	1.4718	1.6298	1.6670	1.7353	1.9394	*G*_5_ ≻ *G*_4_ ≻ *G*_3_ ≻ *G*_2_ ≻ *G*_1_
*P*^Ω^ = 0.3	1.4895	1.6206	1.6490	1.7641	1.9105	*G*_5_ ≻ *G*_4_ ≻ *G*_3_ ≻ *G*_2_ ≻ *G*_1_
*P*^Ω^ = 0.4	1.5116	1.6143	1.6342	1.7940	1.8825	*G*_5_ ≻ *G*_4_ ≻ *G*_3_ ≻ *G*_2_ ≻ *G*_1_
*P*^Ω^ = 0.5	1.5384	1.6112	1.6223	1.8251	1.8553	*G*_5_ ≻ *G*_4_ ≻ *G*_3_ ≻ *G*_2_ ≻ *G*_1_
*P*^Ω^ = 0.6	1.5701	1.6110	1.6134	1.8575	1.8790	*G*_5_ ≻ *G*_4_ ≻ *G*_3_ ≻ *G*_2_ ≻ *G*_1_
*P*^Ω^ = 0.7	1.6072	1.6139	1.6273	1.8911	1.9834	*G*_5_ ≻ *G*_4_ ≻ *G*_3_ ≻ *G*_2_ ≻ *G*_1_
*P*^Ω^ = 0.8	1.6000	1.6197	1.6340	1.9260	1.9787	*G*_5_ ≻ *G*_4_ ≻ *G*_3_ ≻ *G*_2_ ≻ *G*_1_

### 6.7 Comparative analysis

We compared these methodologies thoroughly to assess the efficacy of different decision-making approaches within IFNs. Our findings are more credible and consistent because we utilized robustness and validation tests continuously throughout the research and because we carefully examined each component. There would have been no way for us to conduct our research if these procedure issues hadn’t been addressed. Ultimately, our conclusions are likewise grounded in these criteria. You may find a condensed version of the key results that give a charming synopsis of our study in Table 12. Thanks to the nuanced insights from our systematic investigation, we now have a thorough understanding of the pros and cons of the different decision-making processes utilized inside IFNs. To sum up, our study helps decision-makers with trustworthy insights, guides the strategic integration of IFs, and expands our understanding of decision-making within the IF framework.

**Table 12:** Comparison of newly proposed with already existing when *P*^Ω^ = 0.5.

Authors	Methodology	Ranking of alternatives	Optimal alternative
Kumari and Mishra [70]	COPRAS method	*G*_5_ ≻ *G*_4_ ≻ *G*_3_ ≻ *G*_1_ ≻ *G*_2_	*G*_5_
Rouyendegh [71]	ELECTRE model	*G*_5_ ≻ *G*_4_ ≻ *G*_2_ ≻ *G*_1_ ≻ *G*_3_	*G*_5_
Stanujkić and Karabašević [72]	WASPAS method	*G*_5_ ≻ *G*_2_ ≻ *G*_1_ ≻ *G*_4_ ≻ *G*_3_	*G*_5_
Roy and Garai [73]	delphi method	*G*_5_ ≻ *G*_1_ ≻ *G*_4_ ≻ *G*_2_ ≻ *G*_3_	*G*_5_
Yazdi [74]	TOPSIS method	*G*_5_ ≻ *G*_4_ ≻ *G*_2_ ≻ *G*_3_ ≻ *G*_2_	*G*_5_
Govindan et al. [75]	DEMATEL method	*G*_5_ ≻ *G*_4_ ≻ *G*_1_ ≻ *G*_2_ ≻ *G*_3_	*G*_3_
Proposed	Entropy-CRADIS	*G*_5_ ≻ *G*_4_ ≻ *G*_3_ ≻ *G*_2_ ≻ *G*_1_	*G*_5_

## 7 Limitations and discussion

### 7.1 Limitations

- The availability and high quality of the data are critical to the model’s performance. Getting data on energy use, expenses, emissions, and other factors can be difficult, especially in different industrial contexts. If the data are not correct or full, the model’s results will probably not be true.
- The entropy method assumes that the data offers a complete picture of the decision-maker’s preferences. This technique is used while analyzing criteria. Nevertheless, multiple weighing criteria may arise due to the priorities and viewpoints of the different stakeholders. This subjective element may have an impact on the decision-making process’s results if it is taken into account.
- This method reduces the complexity of the complex systems and interactions seen in smart factories, notwithstanding their complexity. It is possible that an imprecise depiction would fall short of encapsulating the complex and dynamic character of production procedures. This covers the effects of outside variables like market swings and government-enacted legislation.
- IFS are best used when decision-makers fully grasp the idea and how it works. In the process of trying to understand and interpret IFSs, it could be necessary to have access to certain data.
- It’s feasible that the idea will be more or less successful in other sectors and areas. The results and how the suggested method is implemented are very susceptible to the impact of local conditions, legal frameworks, and technological infrastructures.
- It’s probable that resources like money, time, and expertise will need to be allocated to carry out the data collecting, employee training, and integration of state-of-the-art technological processes. This might be a problem for businesses with more constrained budgets.

### 7.2 Discussion

This study uses an MCDM approach and an IF environment to maximize energy efficiency in smart factories. The section under discussion includes an overview of the study’s conclusions and implications. One of the study’s many contributions to the field of methodology is the development of a solid framework for decision-making that combines IFS with MCDM procedures. This framework offers an approach for determining criteria, entropy-weighted criterion weighing, CRADIS-ranked alternative ranking, and sensitivity analysis. It accomplishes this by methodically tackling the complexity and element of uncertainty in the decision-making process for energy efficiency. Identifying and defining eight essential criteria enables the development of an all-encompassing assessment framework that considers pertinent sustainability and energy efficiency areas. The assessment parameters encompass operating expenses, greenhouse gas emissions, energy usage, resource efficiency, technological integration, renewable energy utilization, production effectiveness, and worker well-being.

To continue meeting the strict requirements set out by environmental regulations, businesses urgently need to lower their operational costs and carbon footprint. This study aims to address this need. Energy usage may be reduced by adopting smart factories, which utilize cutting-edge technology like AI and the IoT. However, evaluating and choosing the best choices for increasing energy efficiency is not always easy for those in charge of making decisions. This article offers a framework to help producers overcome these obstacles by assisting them to make decisions logically. The model presented in Section 4 concluded that the best course of action for enhancing smart factories’ energy efficiency was alternative 5. The offered case studies or simulations, which show how precisely the algorithm assesses the various ways to improve energy efficiency, corroborate these results. Sensitivity analysis contributes to the decision-making process’s increased credibility by shedding light on the process’s resilience and the impact of uncertainty. In addition to examining its direct use, the article also discusses the broader implications for environmentally friendly production techniques and the incorporation of cutting-edge technology. The framework that has been suggested helps manufacturers achieve their sustainability objectives and stay in compliance with any regulations by coordinating energy efficiency initiatives with economic and environmental goals. At the end of this section, we shall offer some suggestions for future research directions that the field may pursue. These include extending the framework to incorporate dynamic criteria, including more sophisticated AI tools for predictive analytics, and researching its use in different industries and countries.

## 8 Conclusion and implication

The study presents a sound framework for decision-making to maximize the energy efficiency of smart factories. The system uses MCDM techniques with IFS. Several significant issues are addressed by our plan, such as the mitigation of carbon footprints, efficient control of operating costs, and compliance with strict environmental standards. We have offered a thorough assessment approach that considers the complexities of sustainable manufacturing and energy efficiency by developing and evaluating eight fundamental criteria for using renewable energy, production efficiency, and worker health and safety. The strategy has proven successful in assessing and prioritizing the numerous opportunities for increasing energy efficiency by applying these techniques. Sensitivity analysis, the CRADIS methodology for alternative ranking, and the entropy method for criteria weighting are employed in this process. The adaptability and efficacy of proposed methodology are demonstrated by the results of our empirical investigation. The model identified that alternative 5 best enhances smart factories’ energy efficiency.

### 8.1 Future directions

1. Make sure to include dynamic criteria in the framework construction process so that it may be adjusted to account for changing conditions and production process alterations. Making the decision-making model more responsive could involve a combination of real-time data analytics and machine learning.
2. To enhance these abilities, look at how smart industrial energy management and forecasting may use cutting-edge AI and predictive analytics. AI-driven predictive maintenance algorithms and demand forecasting might be two of these methods.
3. It is crucial to look at the framework’s suitability in various regional and industrial contexts to account for regional energy policy, resource availability, and technical infrastructures.
4. Sensitivity analysis techniques should be enhanced to better understand how assumptions and uncertainties affect decision-making. Risk and variability evaluation may be achieved using probabilistic models and scenario analysis.
5. To encourage greater stakeholder participation, interactive decision-support technology should be used to enhance cooperative decision-making processes. You can ensure all stakeholders have access to the tools by doing a usability study.
6. To increase production sustainability, decrease waste output, and boost resource efficiency, think about researching the possibilities of combining the framework with concepts from the circular economy.
7. It is essential to carry out further case studies and empirical validation of the framework to guarantee that it performs as intended across a range of operational situations and sectors.
