# Correction: Integrating intuitionistic fuzzy and MCDM methods for sustainable energy management in smart factories

**DOI:** 10.1371/journal.pone.0322355

**Published:** 2025-04-04

**Authors:** Mohd Anjum, Naoufel Kraiem, Hong Min, Yousef Ibrahim Daradkeh, Ashit Kumar Dutta, Sana Shahab

The email address for the corresponding author, Hong Min, is incorrect. The correct email address is hmin@gachon.ac.kr.

The ORCID iD is missing for the third author. Please see the author’s ORCID iD here:

- Author Hong Min’s ORCID iD is: 0000-0002-9099-0890 (https://orcid.org/0000-0002-9099-0890).
